# Rapid identification of *Mycobacterium tuberculosis *infection by a new array format-based surface plasmon resonance method

**DOI:** 10.1186/1556-276X-7-180

**Published:** 2012-03-08

**Authors:** Shang-Chen Hsieh, Chia-Chen Chang, Chia-Chen Lu, Chia-Fong Wei, Chuan-Sheng Lin, Hsin-Chih Lai, Chii-Wann Lin

**Affiliations:** 1 Graduate Institute of Medical Biotechnology and Laboratory Science, and Research Center for Pathogenic Bacteria, Chang Gung University, No. 259, Wen-Hwa 1st Road, Kwei-Shan, Tao-Yuan, 333, Taiwan; 2 Institute of Biomedical Engineering, National Taiwan University, No. 1, Sec. 4, Roosevelt Road, Taipei, 10617, Taiwan; 3 Department of Respiratory Therapy, College of Medicine, Fu Jen Catholic University, No. 510, Zhongzheng Road, Xinzhung District, New Taipei City, 24205, Taiwan; 4 Center for Emerging Material and Advanced Devices, National Taiwan University, No. 1, Sec. 4, Roosevelt Road, Taipei, 10617, Taiwan

**Keywords:** identification, TB, SPR, biosensor, antigen

## Abstract

Tubercle bacillus [TB] is one of the most important chronic infectious diseases that cause millions of deaths annually. While conventional smear microscopy and culture methods are widely used for diagnosis of TB, the former is insensitive, and the latter takes up to 6 to 8 weeks to provide a result, limiting the value of these methods in aiding diagnosis and intermediate decisions on treatment. Therefore, a rapid detection method is essential for the diagnosis, prognosis assessment, and recurrence monitoring. A new surface plasmon resonance [SPR] biosensor based on an array format, which allowed immobilizing nine TB antigens onto the sensor chip, was constructed. Simultaneous determination of multiple TB antibodies in serum had been accomplished with this array-based SPR system. The results were compared with enzyme-linked immunosorbent assay, a conventional immunological method. Array-based SPR showed more advantages in providing label-free and real-time detection. Additionally, the high sensitivity and specificity for the detection of TB infection showed its potential for future development of biosensor arrays for TB diagnosis.

## Background

*Mycobacterium tuberculosis *[MTB] is the causative agent of tubercle bacillus [TB], accounting for approximately two million deaths annually, mainly in developing countries [1], and remains one of the leading causes of respiratory infections and has posed critical threats to public health [2]. Currently, the global number of TB cases is rising at a rate of 2% per year [3]. Hence, the key to the control of this infectious disease is to provide the short course therapy and the post-exposure vaccine. Moreover, the rapid detection method with high sensitivity and specificity is essential to aid the diagnosis, assess the prognosis, and monitor the disease recurrence [4].

Until now, many analytical methods have been applied to the routine detection of MTB, which include the staining of acid-fast bacilli [AFB], cultivation and numerous serological and biochemical tests such as polymerase chain reaction [PCR] and enzyme immunoassay [EIA] for identification [5-8]. AFB has been evaluated on sputum samples, giving simple operation but relatively poor sensitivity [9]. Although cultivation usually provides reliable and accurate results, it requires several weeks to obtain a result due to the slow-growing nature of these mycobacteria [10]. PCR-based methods are useful techniques for amplification of small amounts of genetic material but require complicated sample prepurification before analysis. EIA employing multiple antibody probes for bacteria detection leads to both the complexity and the cost of the method. Hence, there are still needs to develop better technologies that can reduce detection complexity and perform faster diagnosis while maintaining high sensitivity and specificity. The uses of nanoparticles and electrochemical and optical methods for nucleic acid detection have been explored extensively [1,11,12]. Other strategies based on antibody-antigen recognition with fluorescence and microgravimetric techniques for analyses of MTB were reported recently [13]. In contrast to the detection of antigen, the bioassay based on antibody detection is an alternative approach for latent TB. Detection of expressed TB polyclonal antibodies is more useful than detection of the monoclonal antibodies since such an antibody may not be expressed in TB-infected individuals, resulting in the poor performance of the TB detection [14]. A multiantigen print immunoassay has been recently employed for profiling multiple antibodies to tuberculosis [15]. Nonetheless, this immunoassay requires longer incubation time to carry out the antibody-antigen interaction compared with other biosensors.

Surface plasmon resonance [SPR] has attracted much attention because of several important properties. The main advantage of an SPR-based assay is that it is an extremely sensitive optical sensor, capable of detecting subnanogram levels in real time without any specific label [16]. Moreover, a SPR biosensor can detect trace amounts of specific analytes from complex fluids without sample preparation [17]. Due to these advantages, SPR has emerged as a powerful optical tool that can greatly provide valuable information on biomedical and chemical analyses [16-20]. In addition, several groups have developed multispot SPR for studying the biomolecular interaction with an array format [21-23]. This technique provides a possible means of quick and simultaneous detection for observing many interaction events and is thus considered as a promising technique for proteome profiling methods. However, to our knowledge, no attempt has been reported to use the SPR-based biosensor for clinical antibody detection of TB diseases in a parallel manner.

In the present study, we developed a new SPR-based biosensor for clinical antibody detection of TB diseases in a parallel manner for the first time. The sensitivity, specificity, and reproducibility of the array-based SPR results were evaluated and finally compared to those of enzyme-linked immunosorbent assay [ELISA], a conventional immunological method.

## Materials and methods

### Chemicals and materials

*N*-hydroxysuccinimide [NHS], 8-mercaptooctanoic acid (C_8_H_16_O_2_S) [8-MOA], phosphate-buffered saline [PBS], 1-ethyl-3-(3-dimethylaminopropyl)carbodiimide hydrochloride [EDC], bovine serum albumin [BSA], and ethanolamine were purchased from Sigma (St. Louis, MO, USA) and used as received. The nine purified antigens were listed in Table 1 and were provided by the National Taiwan University Hospital (Taipei, Taiwan). A total of 57 serum samples from different individuals (*n *= 57) was evaluated. The serum samples of TB patients (*n *= 29) and of healthy individuals (*n *= 28) were obtained from the National Taiwan University Hospital. All healthy individuals were detected negative of TB infection by traditional MTB diagnostic methods and medical history.

**Table 1:** MTB antigens used in the study which were previously purified in the laboratory

Antigen number	Category
W06	Secreted protein
W10	Secreted protein
W14	Heat shock protein
W19	Lipoprotein
W28	Secreted protein
W38	Lipoprotein
W64	Secreted protein
W70	Secreted protein
W85	Fibronectin-binding protein

### Array chip preparation

**Figure 1:**
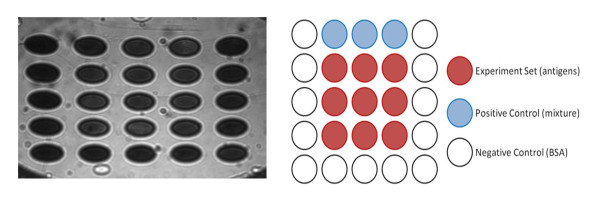
**On-chip TB screening using SPR**. SPR image of the microarrayed chip (left) and the configuration of the SPR array chip (right). Numbers shown in the experiment set indicate the molecular weight of each purified MTB antigen. Positive control means the mixture of MTB antigens used for antibody challenge from rabbits. The BSA was used as the negative control.

Twenty-five-spot protein arrays were designed and fabricated by thin film deposition and photolithography. The sizes of arrays were 2 mm in diameter, and the center-to-center spacing between all adjacent spots was set to 3 mm. 8-MOA in ethanol was dripped onto the array surface at room temperature and allowed to form a self-assembled monolayer for 30 min. The array surface was further treated with 400 mM EDC/100 mM NHS for 10 min to active carboxyl groups of 8-MOA. The nine different antigens, at a concentration of 50 μg/mL, were then applied to the spots of the protein arrays for 1 h, and the arrays were blocked with 1 M ethanolamine for 10 min. The BSA was used as the negative control and did not show significant binding reaction to patients' serum antibodies. The mixture of nine MTB antigens previously purified in the laboratory was deposited in one spot and used as the positive control (Figure 1 and Table 1). The chips were rinsed with PBS buffer and then stored at 4°C for further use.

### SPR measurements

SPR measurements were conducted using the GWC SPRimager system (GWC Technologies, Madison, WI, USA) as described elsewhere [17]. Briefly, the images were observed at the fixed angle which was slightly smaller than the SPR angle and collected with a CCD camera at 790 nm. The SPR signal data were collected with a V++ 4.0 program (Digital Optics, Auckland, New Zealand). The measured response values are expressed as Δ*R*, which represented the normalized reflectivity change. All experiments were carried out at room temperature, and a flow rate of 30 μL/min was used.

### Statistical analysis

The mean value and standard deviation [SD] were calculated using the Sigmaplot 10.0 (Systat Software, Inc., Chicago, IL, USA). The threshold value was determined from the mean value plus twofold SD from the healthy group. This threshold value was used to classify individuals as positive or negative of the TB infection. Detection sensitivity, specificity, positive predictive value [PPV], and negative predictive value [NPV] were calculated by standard statistical methods [24].

## Results and discussion

### SPR-based immunoassays for TB detection

**Figure 2:**
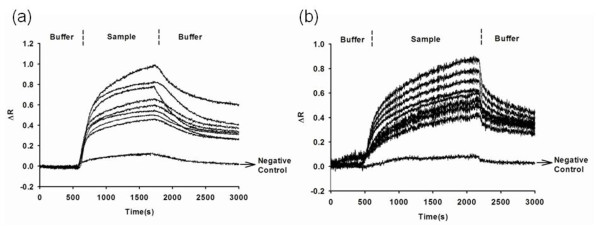
**SPR sensograms correspond to the injection of 10-fold diluted sera into an array-format SPR sensor**. (**a**) SPR results from TB patient sera and (**b**) SPR results from normal control sera. All experiments were carried out at room temperature in triplicate, and a flow rate of 30 μL/min was used. Results shown were averages of three independent experiments.

Multiplex detection of TB antibody was accomplished by combining nine immunoassays onto one sensor chip. Serum specimens from TB patients were distinguishable from control specimens of healthy individuals. Figure 2 showed the real-time SPR binding curves in the injection of sample, while the PBS buffer was used for the SPR running buffer. These results were obtained using the V++ software to acquire relative quantitative reflectivities for the array spots. To properly normalize the reflectance values, the maximum signal was assigned a mean value of 1. Normalizing on the maximum SPR responses was to compare different array chips. Injection of a serum sample of a TB patient, but not a healthy donor, caused an initial increase and then the stabilization of the signal response corresponding to the spot bearing the TB antigen (Figure 2). This sample induced a significant response in reflectivity (*R *> 0.6) from the W14 spot, revealing the presence of a specific antibody against this antigen in the patient-derived serum. Lower reflectivities (0.2 to 0.5) from all spots were observed in the use of healthy donors under the same condition. Additionally, the reflectivities of the negative control experiments were typically lower than 0.02. Thus, the detection signal, reflectivity, is at least two orders-of-magnitude higher than in the negative control. The SPR reflectivity from the healthy individual did increase slightly, illustrating that there might be a small amount of the target antibody for the antigen spots relative to the negative control. However, these levels of antibodies against each antigen in TB patients were higher than those in healthy controls.

**Figure 3:**
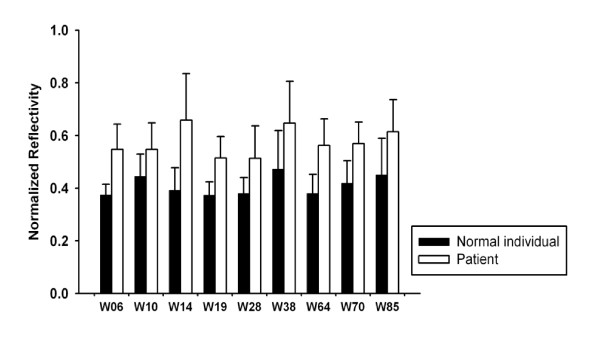
**Comparison of the results**. Comparison of the results obtained from serum samples of normal individuals and patients determined by SPR array-based screening assay. Results were averages of three independent experiments (*p *< 0.05).

Antibodies from patient samples on our SPR array were validated for functionality by interaction with the antigen immobilized on the sensor array. In most cases, signals from normal control were very low, and positive array signals from the patient sample were strong and easily identifiable. However, in our study, the SPR response had a slight increase from the healthy individual (Figure 3). Thus, when discriminating between TB and non-TB patients, it was necessary to set a threshold for detection of the serum sample. Although a higher threshold value would mean fewer false positive results, false negative values would increase. By contrast, lower cutoff threshold had the opposite effect. To determine a cutoff value for a positive result, the SPR reflectivities of the six normal individuals were averaged for this SPR sensor reference. Here, 95% confidence intervals for the SPR test were chosen as the cutoff threshold. Therefore, a threshold value of 0.58 (mean + twofold SD) was used to identify TB infection.

### Diagnostic assessment of the SPR array

The sensitivity, specificity, PPV, and NPV of the SPR-based antigen array were compared with those of the commercial ELISA tests. Serum from 29 TB patients and 28 healthy individuals were evaluated by the use of this cutoff value. As shown in Table 2, the specificity of the SPR-based sensor was all above 90% (93% to 100%), whereas the sensitivity ranged from 14% to 79%. On the other hand, ELISA exhibited similar results with the specificity (79% to 100%) but had a weaker sensitivity (10% to 62%). These results demonstrated that the use of the SPR sensor had the potential to discriminate between TB and non-TB patients.

**Table 2:** Results of sensitivity, specificity, PPV, and NPV of SPR array compared to ELISA assay results

Antigen number^a^	Sensitivity (%)^b^		Specificity (%)^b^		PPV (%)^b^		NPV (%)^b^	
	SPR	ELISA	SPR	ELISA	SPR	ELISA	SPR	ELISA
W06	79	10	96	100	96	100	82	52
W10	45	38	100	96	100	92	64	60
W14	59	24	100	96	100	88	70	55
W19	31	62	96	79	90	75	57	67
W28	86	34	93	96	93	91	87	59
W38	79	24	100	100	100	100	82	56
W64	69	17	100	93	100	71	76	53
W70	14	24	100	100	100	100	53	56
W85	76	28	96	96	96	89	79	56
Multi-Ag	100	90	89	57	91	68	100	84

^a^A total of nine *M. tuberculosis *related antigens were used for the test. ^b^Results shown were averages of three independent experiments. PPV, positive predictive value; NPV, negative predictive value; SPR, surface plasmon resonance; ELISA, enzyme-linked immunosorbent assay; Ag, antigen.

Combination of antigens for TB diagnosis could be superior to the use of a single, antigen-based assay [25,26]. Thus, the sensitivity and specificity of the multiple-antigen ELISA system was compared to those of the SPR array system. As shown in Table 2, the multiple-antigen test provided more accuracy in detecting TB, increasing sensitivity of diagnosis, and facilitating interrogation of sample components simultaneously. The results in the array-based SPR sensor were similar to those obtained for the multiple-antigen ELISA assay. When comparing the sensitivity and specificity in the diagnosis of TB patients, the multiple-antigen strategies for SPR and ELISA systems performed better than the single-antigen tests for those assays. However, the performance of this multiplexed measurement for SPR sensor was still higher than that of multiple-antigen ELISA in terms of sensitivity (100%, 90%), specificity (89%, 57%), PPV (91%, 68%), and NPV (100%, 84%). Hence, this SPR-based antigen array has great potential in the rapid identification of TB infection.

## Conclusion

Simultaneous determination of nine TB antibodies in serum had been accomplished with an array-based SPR system. In comparison with ELISA using enzyme or secondary antibody as a single label, SPR was more advantageous in providing label-free and real-time detection. Additionally, this SPR-based antigen array had high specificity and sensitivity for the detection of TB infection. This assay showed excellent promise for further development of biosensor arrays for multiple antigen detection of TB infection.

## Competing interests

The authors declare that they have no competing interests.

## Authors' contributions

S-CH and C-CC participated in the research design. S-CH, C-CC, C-FW, and C-SL conducted the experiments. H-CL and C-WL contributed the analytic tools. S-CH, C-CC, and C-CL analyzed the data. H-CL and C-WL wrote the paper. All authors read and approved the final manuscript.
